# Clinical Evaluation of Diagnosis Efficacy of Active *Mycobacterium tuberculosis* Complex Infection via Metagenomic Next-Generation Sequencing of Direct Clinical Samples

**DOI:** 10.3389/fcimb.2019.00351

**Published:** 2019-10-18

**Authors:** Xian Zhou, Honglong Wu, Qiaoling Ruan, Ning Jiang, Xinchang Chen, Yaojie Shen, Yi-Min Zhu, Yue Ying, Yi-Yi Qian, Xuyang Wang, Jing-Wen Ai, Wen-Hong Zhang

**Affiliations:** ^1^Department of Infectious Diseases, Huashan Hospital, Fudan University, Shanghai, China; ^2^Tianjin Translational Genomics Center, BGI-Tianjin, Binhai Genomics Institute, BGI-Shenzhen, Tianjin, China; ^3^School of Life Sciences, Fudan University, Shanghai, China

**Keywords:** diagnosis, *Mycobacterium tuberculosis*, tuberculosis, metagenomic next-generation sequencing, Xpert MTB/RIF

## Abstract

**Background:** Tuberculosis (TB) is now the leading cause of death from infectious disease. Rapid screening and diagnostic methods for TB are urgently required. Rapid development of metagenomics next-generation sequencing (mNGS) in recent years showed promising and satisfying application of mNGS in several kinds of infectious diseases. However, research directly evaluating the ability of mNGS in TB infection is still scarce.

**Methods:** We conducted an adult prospective study in mainland China to evaluate the diagnostic performance of mNGS for detection of *Mycobacterium tuberculosis* complex (MTB) in multiple forms of direct clinical samples compared with GeneXpert MTB/RIF assay (Xpert), traditional diagnostic methods, and the clinical final diagnosis.

**Results:** Of 123 patients presenting with suspected active TB infection between June 1, 2017, and May 21, 2018, 105 patients underwent synchronous tuberculous testing with culture, Xpert, and mNGS on direct clinical samples including sputum, cerebrospinal fluids, pus, etc. During follow-up, 45 of 105 participants had clinical final diagnosis of active TB infection, including 13 pulmonary TB cases and 32 extrapulmonary TB cases. Compared to clinical final diagnosis, mNGS produced a sensitivity of 44% for all active TB cases, which was similar to Xpert (42%) but much higher than conventional methods (29%). With only one false-positive result, mNGS had a specificity of 98% in our study. mNGS yielded significantly much higher sensitivity in pre-treatment samples (76%) than post-treatment ones (31%) (*P* = 0.005), which was also true for Xpert and conventional methods. Combining Xpert and mNGS together, the study identified 27 of 45 active TB cases (60%), including all 13 conventional method-identified cases, and the result reached statistical significance compared to conventional methods (McNemar-test *P* < 0.001).

**Conclusions:** mNGS had a similar diagnostic ability of MTB compared with Xpert and showed potential for a variety of clinical samples. Combined mNGS and Xpert showed an overall superior advantage over conventional methods and significantly improved the etiology diagnosis of both MTB and other pathogens. The result that anti-TB treatment significantly reduced diagnostic efficacy of culture, Xpert, and mNGS highlighted the importance of collecting samples before empirical treatment.

## Introduction

Tuberculosis (TB) is now the leading cause of death from infectious disease, killing people even more than HIV and malaria worldwide (World Health Organization, 2017). An important reason for TB leading to so many deaths is the difficulty of diagnosis, with 40% of estimated incident cases failing to be identified and reported (World Health Organization, 2017). The GeneXpert MTB/RIF assay (Cepheid Inc., Sunnyvale, CA, USA) (hereinafter referred to as “Xpert”) was endorsed by the World Health Organization in 2010. However, it has not improved global case detection rates and showed limited efficacy in extrapulmonary TB (World Health Organization, 2013). As rapid and accurate diagnosis of TB is a prerequisite for effective treatment, alternative rapid screening and diagnostic methods are urgently required.

Recent years have witnessed rapid development of metagenomics next-generation sequencing (mNGS), featured with the advantage of shortened turnaround time, unbiased detection, and semi-quantitative value in follow-up. To date, several studies have addressed the value of mNGS in finding out the causative pathogens and guiding targeted antimicrobial therapy more quickly (Wilson et al., 2014, 2015, 2017, 2018; Guan et al., 2016; Kawada et al., 2016; Wüthrich et al., 2016; Wylie et al., 2016; Chiu et al., 2017; Murkey et al., 2017; Piantadosi et al., 2017), and a myriad of intense researches have reported their satisfying application of mNGS in several kinds of infectious diseases including central nervous system infection (Wilson et al., 2014, 2018; Brown et al., 2018), prosthetic joint infection (Simner et al., 2018), bloodstream infection (Long et al., 2016; Gosiewski et al., 2017), respiratory tract infection (Langelier et al., 2018), etc. However, research directly evaluating the ability of mNGS in pulmonary TB and extrapulmonary TB is still scarce; most previous studies have only focused on clinical cases. Therefore, we conducted a single-center prospective study in mainland China to analyze the diagnostic performance of mNGS for detection of *Mycobacterium tuberculosis* complex (MTB) in multiple forms of direct clinical samples in adults compared with Xpert, conventional diagnostic methods including culture and biopsy, and the clinical final diagnosis.

## Materials and Methods

### Study Participants

**Figure 1:**
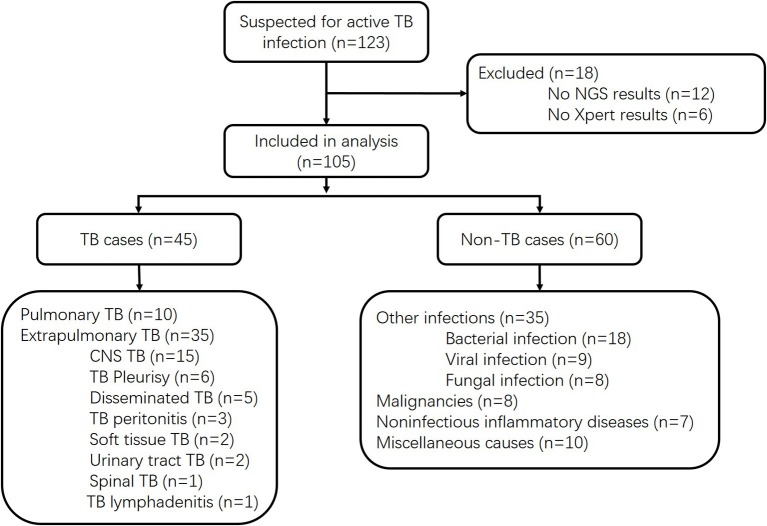
Study design.

We prospectively screened all patients admitted into the Huashan Hospital with suspicion of active TB infections, including both pulmonary TB and extrapulmonary TB, by his or her attending physician from June 1, 2017, to May 21, 2018. As a tertiary hospital, Huashan Hospital admitted patients from Shanghai and all over the country who were referred from local hospitals. Samples were prepared and gathered from suspected infected parts of enrolled patients, and all clinical specimens were immediately sent for culture, Xpert, mNGS, and other microbiological assays if necessary (Figure 1). Samples were non-duplicated from the same patient. The volume and preparation of the samples were carried out in accordance with the requirements of each test, and recommendation for Xpert sampling in this study was listed in the Supplemental Material. Conventional methods refer to the TB culture (Supplemental Material) for fluid specimen or pathological methods for tissue samples. Specimens for Xpert were processed following the manufacturer's instructions. Ethical approval was achieved from Huashan Hospital ethical committee. Informed consents were signed by patients or surrogates. Exclusion criteria included (1) incomplete clinical data, (2) incomplete microbiological data, and (3) insufficient specimen for mNGS. Clinical final diagnoses of the enrolled cases were made by the attending physicians according to China Clinical Treatment Guide for Tuberculosis (Association, 2005) and other clinical guidelines at least 3 months after the specimen collection. TB cases included microbiologically confirmed TB cases and clinically diagnosed TB cases. Patients with MTB culture-positive or Xpert-positive results for MTB would be classified as microbiologically confirmed TB cases. For patients without microbiological evidence, the attending physician may only clinically diagnose active MTB infection by combining the patient's clinical manifestations and imaging findings to exclude other diseases, together with the patient's confirmed responsiveness to anti-TB treatment after 1 month of follow-up. The non-MTB group included infections other than TB, malignancies, non-infectious inflammatory diseases, and miscellaneous causes.

### Sample Processing and Library Construction

A 300-μl sample of cerebrospinal fluid (CSF), bronchoalveolar lavage fluid (BALF), pleural effusion, ascites, etc. were directly collected in DNase-free tubes for the identification of potential pathogens, while tissue, urine, and sputum required a special procedure before nucleic acid extraction: the tissue sample was ground into homogenate, the urine was concentrated, and the sputum was liquefied. A common issue in metagenomic sequencing is the introduction of contaminating microbial nucleic acid during sample preparation. Considering the limitation of labor and fund, we did the whole operation procedure once a day, so all the samples were tested for mNGS immediately or stored in −20°C <24 h before that. The potential contaminating source includes PCR reagents, nucleic acid extraction kits, human skin, as well as environment. In order to control the effect of contamination, a negative control was prepared in parallel and sequenced in the same run.

DNA was extracted with TIANamp Micro DNA Kit (DP316, TIANGEN BIOTECH, Beijing, China) and QIAamp Viral RNA Mini Kit (52906, Qiagen, China) following the manufacturer's operational manual. In addition to plasma, the DNA/cDNA from other samples was fragmented using Bioruptor Pico instrument to generate 200- to 300-bp fragments (Bioruptor Pico protocols). Then, the libraries were constructed as follows: first, the DNA fragments were subjected to end repair and added A-tailing in one tube; subsequently, the resulting DNA was ligated with bubble adapters, which contained barcode sequence, and then amplified by the PCR method. Quality control was carried out using a bioanalyzer (Agilent 2100, Agilent Technologies, Santa Clara, CA, USA) to assess the DNA concentration and fragment size. Qualified libraries were pooled together to make a single-strand DNA (ssDNA) circle and then generated DNA nanoballs (DNBs) by rolling circle replication (RCA). The final DNBs were loaded into a sequence chip and sequenced on the BGISEQ platform (MGI BGISEQ-50, BGI-Wuhan Clinical Laboratories, BGI-Shenzhen, Wuhan 430074, China) in Huashan Hospital using 50-bp/single-end sequencing. Finally, the optical signals were collected using a high-resolution imaging system, and then the optical signals were transformed into digital information, which can be decoded into DNA sequence information.

### Bioinformatics Analysis

All raw reads were quality filtered using an in-house program, including filtering adapter contamination, low-quality, and low-complexity reads. Next, the clean reads after quality filtering were mapped to a human reference database including hg38 and Yanhuang genome sequence using Burrows–Wheeler Alignment (Version: 0.7.10). The remaining reads were aligned to the non-redundant bacterial, virus, fungal, and parasite databases using Burrows–Wheeler Alignment (Version: 0.7.10). The mapped data were processed for advanced data analysis.

We downloaded all the reference genome from the public database, such as NCBI (ftp://ftp.ncbi.nlm.nih.gov/genomes/). Currently, our databases contain 4,152 whole genome sequence of viral taxa, 3,446 bacterial genomes or scaffolds, 206 fungi related to human infection, and 140 parasites associated with human diseases. The reference genome of *M. tuberculosis* is NC_00962.3. The depth and coverage of each species were calculated with the SOAPCoverage software from https://github.com/aquaskyline/SOAPcoverage. The parameter values were normalized according to the data size, which was 8 million reads for sputum and bronchoalveolar lavage fluid, and 20 million reads for other samples. The detected species that existed in suspected background database or/and was also detected in negative control sample was filtered, if they reached the threshold (Li and Durbin, 2009).

### Criteria for a Positive mNGS Result

As previously described (Zhang et al., 2019), the sequencing data of each sample are categorized into four tables each representing bacteria, fungi, virus, and parasite, and one complete table was merged.

Bacteria (mycobacteria excluded), virus, fungi, and parasites: mNGS identified a microbe (species level) whose read number was in the top 10 in the complete belonging list. We identified the causative pathogen according to the pipeline we established before (Zhang et al., 2019).

Mycobacteria: MTB was considered positive when the mapping read number (genus or species level) was in the top 20 in the bacteria list (Miao et al., 2018).

### Statistical Analysis

Continuous variants were described by means when they conform to the *t*-test. Fisher's exact-test was used to evaluate independent binomial variables. Sensitivity and specificity of different methods were assessed. The results were presented with the range of 95% confidence intervals. We did analysis of variance test to compare differences across subgroups. Statistical analyses and figures were conducted using the SPSS statistical package 12.0 software and GraphPad Prism 5 software.

## Results

Of 123 patients presenting with suspected active TB infection between June 1, 2017, and May 21, 2018, 105 patients underwent tuberculous testing with culture, Xpert, and mNGS on direct clinical samples (Figure 1, Table S1). Eighteen patients were excluded because their limited specimen was not enough for all three tests and the priority test was chosen by their attending physician. Forty-five of one hundred five participants had clinical final diagnosis of active TB infection, including 13 pulmonary TB cases and 32 extrapulmonary TB cases. Patient characteristics of those with and without active TB infection did not differ aside from lower white blood cell count and higher proportion with positive blood T.SPOT *TB* results (Table 1).

**Table 1:** Baseline characteristics of the study population.

	**TB (*n* = 45)**	**Non-TB (*n* = 60)**
**Age (mean), years**	49.3	46.3
**Sex—male**, ***n*** **(%)**	27 (60%)	45 (75%)
**Laboratory parameters**		
Hemoglobin (mean), g/L	115.4	116.3
WBC (mean)^*^, 10^9^/L	7.2	9.1
T.SPOT *TB* (+)^**^, *n* (%)	37 (82.2%)	20 (33.3%)
**Pulmonary samples**, ***n***		
Sputum	2	2
BALF	11	11
Lung biopsy tissue	0	1
**Extrapulmonary samples**, ***n***		
CSF	16	33
Pleural fluid	6	5
Ascites	3	5
Pus	5	2
Synovial fluid	0	1
Urine	2	0
**ATT prior to sample collection**, ***n***	16	11

*WBC, white blood cell count; CSF, cerebrospinal fluid; BALF, bronchoalveolar lavage fluid; ATT, anti-TB treatment*.

* *P = 0.0278*,

** *P < 0.0001, Fisher's exact test*.

We assessed diagnostic performance for mNGS, Xpert, and traditional methods compared with the clinical final diagnosis (Table 2). mNGS produced a sensitivity of 44% for all active TB cases, which was similar to Xpert (42%) but much higher than conventional methods (29%). Diagnostic performance varied according to specimen type. In 13 pulmonary TB cases, mNGS reached the same sensitivity of 62% as Xpert, which was also superior to conventional methods (38%). In 16 CSF samples of clinical diagnosed tuberculous meningitis patients, mNGS had a sensitivity of 44%, which was much higher than either Xpert (19%) and conventional methods (19%). mNGS produced one false-positive case of MTB in a CSF sample (Patient no. 93, Table S1), which was negative with culture, Xpert, and PCR of MTB in residual sample. The patient was diagnosed as having autoimmune encephalitis in the end. It was also the only false-positive result of MTB in the whole cohort. While for other extrapulmonary TB samples, including six pleural fluid samples, five pus samples, three ascites samples, and two urine samples, Xpert identified eight cases with a sensitivity of 50%, which outperformed both mNGS (31%) and conventional methods (31%).

**Table 2:** Diagnostic performance of MTB for mNGS, Xpert, and traditional methods compared with the clinical final diagnosis.

	**Sensitivity (95% CI; *n*/*N*)**	**Specificity (95% CI; *n*/*N*)**
**All samples**		
Conventional	29% (0.16–0.44; 13/45)^*^	100% (0.94–1; 60/60)
Xpert	42% (0.28–0.58; 19/45)	100% (0.94–1; 60/60)
mNGS	44% (0.30–0.60; 20/45)	98% (0.91–1; 59/60)
Xpert and mNSG	60% (0.44–0.74; 27/45)^*^	98% (0.91–1; 59/60)
**Pulmonary samples**		
Conventional	38% (0.14–0.68; 5/13)	100% (0.77–1; 14/14)
Xpert	62% (0.32–0.86; 8/13)	100% (0.77–1; 14/14)
mNGS	62% (0.32–0.86; 8/13)	100% (0.77–1; 14/14)
Xpert and mNSG	69% (0.39–0.91; 9/13)	100% (0.77–1; 14/14)
**CSF samples**		
Conventional	19% (0.04–0.46; 3/16)	100% (0.89–1; 33/33)
Xpert	19% (0.04–0.46; 3/16)	100% (0.89–1; 33/33)
mNGS	44% (0.2–0.7; 7/16)	97% (0.84–99; 32/33)
Xpert and mNSG	56% (0.30–0.80; 9/16)	97% (0.84–99; 32/33)
**Other extrapulmonary samples**		
Conventional	31% (0.11–0.59; 5/16)	100% (0.75–1; 13/13)
Xpert	50% (0.25–0.75; 8/16)	100% (0.75–1; 13/13)
mNGS	31% (0.11–0.59; 5/16)	100% (0.75–1; 13/13)
Xpert and mNSG	56% (0.30–0.80; 9/16)	100% (0.75–1; 13/13)

* *The combined sensitivity of Xpert and mNGS reached statistical significance compared to conventional methods (McNemar-test P < 0.001)*.

**Figure 2:**
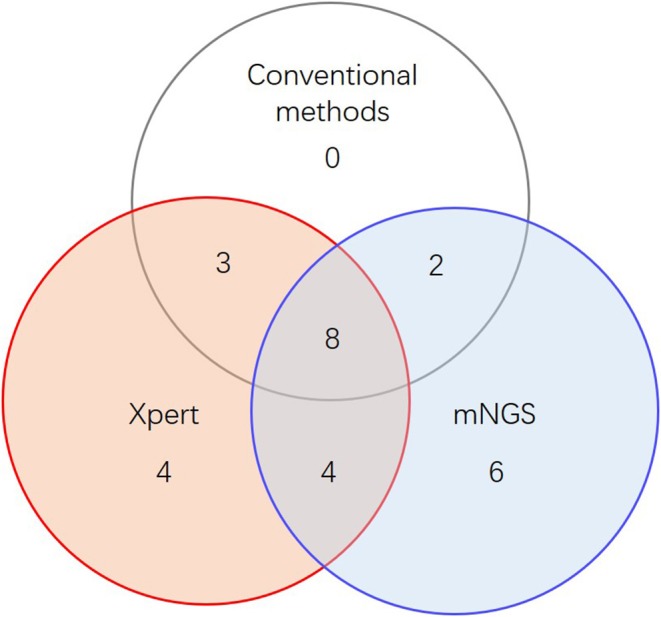
Venn diagram of overlap in active TB diagnostics.

When combining Xpert and mNGS, the two rapid diagnostic methods together, we found that it identified 27 of 45 active TB cases (Figure 2, Table 2), including all 13 conventional method-identified cases, and reached statistical significance compared to conventional methods (McNemar-test *P* < 0.001). The combined diagnostic efficacy of Xpert and mNGS also outperformed any of the three methods in all types of samples (Table 2).

The efficacy of culture, Xpert, and mNGS was significantly affected by anti-TB treatment, as pre-treatment samples yield much higher sensitivity than post-treatment ones, with most of the odds ratio (OR) values more than 1.0 (Table 3). The only exception was in extrapulmonary specimens other than CSF, as two post-treatment specimens positive for all three methods were pus. This indicated the importance of collecting sample before empirical treatment.

**Table 3:** The positive diagnostic results of pre-treatment samples compared to post-treatment ones with conventional methods, Xpert, and mNGS.

	**Post-treatment sensitivity (95% CI; *n*/*N*)**	**Pre-treatment sensitivity (95% CI; *n*/*N*)**	**OR (95% CI)**	*P*-value^*^
**All samples**				
Conventional	13% (0.02–0.38; 2/16)	38% (0.21–0.58; 11/29)	4.28 (0.81–22.52)	0.0943
Xpert	19% (0.04–0.46; 3/16)	55% (0.36–0.74; 16/29)	5.33 (1.24–22.82)	0.0273
mNGS	25% (0.07–0.52; 4/16)	55% (0.36–0.74; 16/29)	3.69 (0.96–14.21)	0.661
Xpert and mNSG	31% (0.11–0.59; 5/16)	76% (0.56–0.90; 22/29)	6.91 (1.78–26.86)	0.005
**Pulmonary samples**				
Conventional	0% (0.0–0.8417; 0/2)	45% (0.17–0.77; 5/11)	4.23 (0.17–108.3)	0.4872
Xpert	0% (0.0–0.8417; 0/2)	73% (0.39–0.94; 8/11)	12.14 (0.46–323.5)	0.1282
mNGS	0% (0.0–0.8417; 0/2)	73% (0.39–0.94; 8/11)	12.14 (0.46–323.5)	0.1282
Xpert and mNSG	0% (0.0–0.8417; 0/2)	82% (0.48–0.98; 9/11)	19.00 (0.67–537.0)	0.0769
**CSF samples**				
Conventional	0% (0.0–0.34; 0/9)	43% (0.10–0.82; 3/7)	14.78 (0.62–351.5)	0.0625
Xpert	11% (0.0–0.48; 1/9)	29% (0.04–0.71; 2/7)	3.20 (0.23–45.2)	0.5500
mNGS	22% (0.03–0.60; 2/9)	71% (0.29–0.96; 5/7)	8.75 (0.90–84.85)	0.1262
Xpert and mNSG	33% (0.07–0.70; 3/9)	86% (0.42–0.99; 6/7)	12.00 (0.96–150.8)	0.0601
**Other extrapulmonary samples**				
Conventional	40% (0.05–0.85; 2/5)	27% (0.06–0.61; 3/11)	0.56 (0.06–5.22)	1.0000
Xpert	40% (0.05–0.85; 2/5)	55% (0.23–0.82; 6/11)	1.80 (0.21–15.42)	1.0000
mNGS	40% (0.05–0.85; 2/5)	27% (0.06–0.61; 3/11)	0.56 (0.06–5.22)	1.0000
Xpert and mNSG	40% (0.05–0.85; 2/5)	64% (0.31–0.89; 7/11)	2.63 (0.30–23.01)	0.5962

* *P-values were calculated with Fisher's exact-test*.

**Figure 3:**
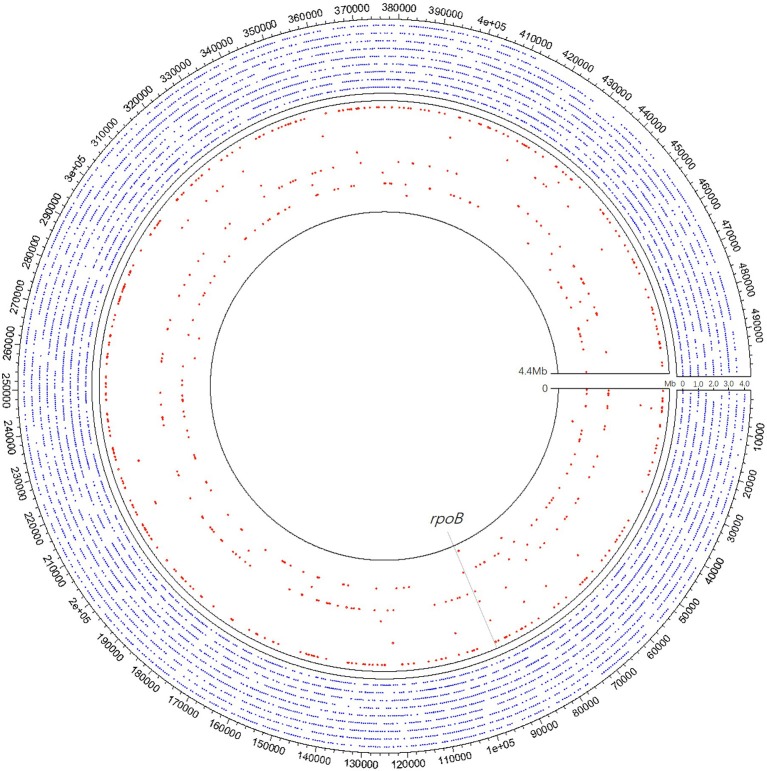
A schematic diagram of mNGS of 10 samples' genomic coverage across *M. tuberculosis* reference genome. The result shows that mNGS was able to detect much more region of the genome apart from *rpoB*. Outer nine circles of the blue dot area all refer to one pus sample of coverage 18.73%, of which each circle represents 0.5 Mb of the genome. Each of the nine red circles of the inner layer represents one sample. The gray dotted line represents the *rpoB* region.

In clinical samples detected MTB positive by mNGS, the coverage across MTB reference genome ranged from 123 bp (0.003%) to 826,259 bp (18.730%), and the detected sequences through mNGS varied among different samples (Figure 3). Xpert discovered two of the specimens positive for rifampicin resistance while detecting 19 cases of TB positive. Although these two rifampicin resistance specimens were not detected by mNGS, in our clinical samples, mNGS recovered regions of *folC, gyrA, gyrB, inhA, katG, rrl*, and *rpsA*, and found S46R mutation in *folC* and A384V mutation in *gyrA*.

Apart from detecting MTB, other pathogens such as *Staphylococcus aureus, Pseudomonas aeruginosa, Human herpesvirus*, etc., were also detected in enrolled specimens (Table S1). In 60 non-TB cases, 16 samples initially suspected to be MTB identified other causative pathogens through mNGS, and all of which were validated through targeted PCR in residual samples and consistent with clinical diagnosis. Two samples positive in mNGS were also positive in culture of *S. aureus* (Patient nos. 62 and 63, Table S1), which further confirmed the mNGS results. In 45 TB cases, mNGS were positive for other pathogens in 8 cases. Considering the clinical diagnosis, of the four cases, we believe that there may be cases of co-infection or colonization (Patient nos. 27, 29, 40, and 45, Table S1), and the other four cases were considered false positive (Patient nos. 3, 9, 12, and 15, Table S1). Because all these mNGS positive cases were validated through targeted PCR in residual samples, these four false positives may be due to contamination acquired throughout sample collection and processing. The most common detected background sequences are listed in Table S1.

We compared the full turnaround time with Xpert, mNGS, and MTB culture, which was from the sample collection to reporting results to clinicians. With processing time around 2 h, the full turnaround time of Xpert in clinical practice was within 24 h. The workflow of mNGS can be broken down into several parts: the sample processing and nucleic acid extraction (7 h), library preparation (7 h), mNGS sequencing (14 h), and data analysis (3 h). Including all the waiting times, we controlled the full turnaround time of mNGS within 72 h, which was longer than Xpert but presented significant advantage over culture (Figure S1).

## Discussion

Although case studies have shown the potential of mNGS in identifying MTB infection (Votintseva et al., 2017) and drug-resistant MTB directly from routinely obtained diagnostic sputum samples (Brown et al., 2015; Doyle et al., 2018), consensus on the mNGS's detection ability for MTB has not been reached. This cohort aimed to apply mNGS in direct clinical samples to evaluating its diagnostic efficacy for active TB infection. We chose to use clinical diagnosis rather than conventional methods as the reference standard, because in our cohort, most of the samples were extrapulmonary specimens suspected for extrapulmonary TB, which is the weakness of conventional methods. In addition, if the conventional method is used as the reference standard, the advantages of other tests over it will not be reflected. Although the clinical diagnosis lacks the evidence of culture or pathological results, the follow-up of more than 3 months greatly reduces the possibility of misdiagnosis. Therefore, we believe that it is a suitable choice to use the clinical diagnosis as the reference standard when comparing the three diagnostic methods.

In terms of TB diagnostic performance, mNGS is not inferior to Xpert in our cohort overall, which is better in CSF and slightly worse in pleural effusion and ascites. Part of the reason may be in the sample collection and processing. The mNGS used about 1 ml of the fluid sample while Xpert's requirement for sample processing prior to machine analysis varied according to the specimen's type and amount. The average testing volume of CSF for Xpert was 3.4 ml, which was probably not enough for its optimal performance (Bahr et al., 2015). For pleural effusion and ascites, the average sample volume used in Xpert was 80 ml before centrifugation. This indicated that mNGS could be more suitable for scarce samples. A centrifuge might increase the detection sensitivity for MTB. One difficulty for mNGS detection of TB may be that the intracellular bacterium, such as MTB, would release fewer extracellular nucleic acids due to its intracellular growth characteristics. mNGS may also detect multiple drug-resistant mutations simultaneously, but it highly relied on coverage. The same principle should be applied to strain identification within the MTB complex. Although mNGS can identify MTB and NTM, due to the high internal homology, it is difficult to determine the species within the MTB complex in the case of insufficient coverage and targeted PCR should be performed to further identify the specific TB strain if necessary. Anti-TB treatment is another influencing factor. It significantly reduced diagnostic sensitivity for all three diagnostic methods, which highlighted the importance of collecting sample before empirical treatment.

Featured with the properties of shortened turnaround time compared to culture and unbiased detection, mNGS is advantageous in testing clinical samples without any prior suspicion of certain pathogens required. In this study, 16 samples of non-TB cases detected pathogens other than MTB, demonstrating the benefit of shotgun metagenomics sequencing in specimens suspected of infections. mNGS also holds the potential in identifying newly emerging and reemerging infections and fueling surveillance and prescribing appropriate antibiotics.

Non-pathogenic microbe sequences detected in our study may come from environmental contamination or reagent pollution. What's more, the high background concentration of a few samples may result from sequencing data from human host, which may correspondingly reduce the data size of pathogen. We used a negative control in every sequencing run to control contamination, and all samples in this study were managed according to the same protocol in the same laboratory.

The false positives of mNGS can be mainly divided into two categories. One type comes from the mNGS procedures, including contaminant pathogen DNA across samples during mNGS library preparation, low-complexity sequences matching low-quality reads from the sample, misannotated species, or contaminants from database entries that also contain reads to human DNA, sequencing adaptors, or vector colonization. We performed targeted PCR in residual samples to confirm the mNGS results and exclude false positivity during the mNGS procedures, and Patient no. 93 with mNGS positive of MTB but negative in PCR was the only false-positive case of this type in our study. The other type of false positive comes from the process before mNGS procedures, mainly from contamination acquired throughout sample collection and processing, which can only be excluded by clinical diagnosis, or resampling if possible. To reduce the risk of contamination introduced before mNGS procedures, we made a strict sterile procedure during sample collection and a relative nucleic acid-free guideline during specimen preparing. In addition, two independent experienced clinics analyzed the mNGS results to see whether it was in accordance with the patient's culture results, clinical manifestation, and diagnosis, and they identified four cases in our cohort that were considered contamination during specimen preparation. The mNGS-detected pathogens of these four cases were all skin colonization or common bacteria in the environment and were all validated in PCR, negative in culture, and inconsistent with clinical manifestations or diagnosis.

Our study has some limitations. First, our study had a relatively small sample size and therefore required more future studies to further investigate the diagnostic value of mNGS in pulmonary and extrapulmonary TB. Second, as a tertiary hospital, the patients admitted were mostly undiagnosed patients after initial treatment, which resulted in our cohort having a larger proportion of post-treatment specimens and in a relatively low overall sensitivity for all diagnostic methods. Also, a Bactec microbial detection system for culture could be applied to raise positivity (Akcam et al., 2006). Further, as the mNGS results may be easily influenced by many factors, the standards in our study should be thoroughly modified and tested before application to other centers, and the false-positive risks should be carefully monitored and evaluated.

## Conclusion

This cohort study demonstrated that in suspected TB-infected patients, mNGS had a similar diagnostic ability of MTB compared with Xpert, and possibly could be more suitable for scarce samples such as CSF. Combined mNGS and Xpert showed an overall superior advantage over conventional methods and significantly improved the etiology diagnosis of both MTB and other pathogens. The result that anti-TB treatment significantly reduced diagnostic efficacy of culture, Xpert, and mNGS highlighted the importance of collecting sample before empirical treatment. In the future, multi-center studies will be needed to explore universal criteria or guidelines of mNGS in active TB infections.

## Data Availability Statement

The raw data supporting the conclusions of this manuscript is available from the corresponding author on reasonable request.

## Ethics Statement

Ethical approval was achieved from Huashan Hospital ethical committee. Informed consents were signed by patients or surrogates.

## Author Contributions

XZ, HW, QR, J-WA, and W-HZ conception or design of the work. XC, YS, Y-MZ, YY, Y-YQ, and XW data collection and patient care. XZ, HW, QR, J-WA, NJ, and XC data analysis and interpretation. XZ, HW, and J-WA drafting the article. J-WA and W-HZ critical revision of the article. XZ, HW, QR, NJ, XC, YS, Y-MZ, YY, Y-YQ, XW, J-WA, and W-HZ final approval of the version to be published.

### Conflict of Interest

The authors declare that the research was conducted in the absence of any commercial or financial relationships that could be construed as a potential conflict of interest.
