# Supplementary material for: Clinical Evaluation of Diagnosis Efficacy of Active Mycobacterium tuberculosis Complex Infection via Metagenomic Next-Generation Sequencing of Direct Clinical Samples
Source: Front Cell Infect Microbiol. 2019 Oct 18;9:351. doi: 10.3389/fcimb.2019.00351 (PMC6813183; doi:10.3389/fcimb.2019.00351)
Supplement: Supplementary file 1 [file Table_1.docx]

**Supplemental materials**

**Sputum decontamination**

Clinical specimens were decontaminated using the N-acetyl-L-cysteine sodium hydroxide method (NALC-NaOH). After centrifugation, the pellet was resuspended in 1 to 1.5 ml of sterile phosphate buffer (pH 6.8). This suspension was used for inoculation of culture media. A smear of the processed sample was prepared and examined for the presence of AFB.

**MTBC culture**

Liquid culture media based on fluorometric detection of growth. Mycobacteria Growth Indicator Tube (MGIT) tubes were inoculated with 0.5 ml of the processed specimen. The tubes were incubated in the MGIT 960 instrument at 37°C.

Solid culture media was inoculated with 0.25 ml suspension processed for each specimen and incubated at 37°C. For tubes identified as positive, a smear of a sample from the tube was prepared for examination for AFB. All smears were stained by the Kinyoun method and examined with a light microscope. MTB strains isolated from culture were identified using the MGIT TBc ID method (MPT 64: Becton Dickinson, Sparks, Maryland, USA).

**Recommendation for Xpert sampling**

Sputum: no food residue

BALF: >2ml

CSF: >3ml

Pleural effusion: >50ml

Ascites: >50ml

Urine: No anti-tuberculosis drugs. Leave 24h urine to mix or take 200ml of sediment.

Pus: >1m

**Figure S1.** Kaplan-Meier curve of MTB positive rate by mNGS, Xpert and culture. * Log-rank test, P<0.0001.

Table S1. The cohort’s diagnostic results.

| Patient No | Sample type | TB diagnosis | Clinical diagnosis | Xpert | Conventional methods | mNGS detected pathogens | | TOP 5 mNGS detected non-pathogenic microorganisms | | | | |
| --- | --- | --- | --- | --- | --- | --- | --- | --- | --- | --- | --- | --- |
|  |  |  |  |  |  | Pathogen-1 | Pathogen-2 | No.1 | No.2 | No.3 | No.4 | No.5 |
| 1 | CSF | TB case | CNS TB | (-) | Negative |  |  | Propionibacterium acnes | Burkholderia 383 | Thermus thermophilus | Staphylococcus epidermidis | Micrococcus luteus |
| 2 | CSF | TB case | CNS TB | (+) | Culture: M. tuberculosis |  |  | Propionibacterium acnes | Bradyrhizobium japonicum | Corynebacterium callunae | Methylobacterium radiotolerans | Pseudomonas TKP |
| 3 | CSF | TB case | CNS TB | (-) | Negative | M. tuberculosis | Pseudomonas putida | Propionibacterium acnes | Corynebacterium callunae | Methylobacterium radiotolerans | Acidovorax KKS102 | Asticcacaulis excentricus |
| 4 | CSF | TB case | CNS TB | (-) | Negative |  |  | Bradyrhizobium S23321 | Burkholderia 383 | Ralstonia solanacearum | Propionibacterium acnes | Malassezia globosa |
| 5 | CSF | TB case | CNS TB | (-) | Negative |  |  | Burkholderia multivorans | Propionibacterium acnes | Ralstonia solanacearum | Gardnerella vaginalis | Acidovorax KKS102 |
| 6 | CSF | TB case | CNS TB | (-) | Negative |  |  | Bradyrhizobium japonicum | Agromonas oligotrophica | Ralstonia solanacearum | Propionibacterium acnes | Mesorhizobium ciceri |
| 7 | CSF | TB case | CNS TB | (-) | Negative |  |  | Bradyrhizobium japonicum | Ralstonia solanacearum | Propionibacterium acnes | Methylobacterium radiotolerans | Nitrobacter hamburgensis |
| 8 | CSF | TB case | CNS TB | (+) | Negative | M. tuberculosis |  | Bradyrhizobium japonicum | Agromonas oligotrophica | Propionibacterium acnes | Acidovorax ebreus | Staphylococcus epidermidis |
| 9 | CSF | TB case | CNS TB | (-) | Tuberculosis PCR positive | M. tuberculosis | Pseudomonas aeruginosa | Propionibacterium acnes | Burkholderia multivorans | Ralstonia solanacearum | Acidovorax KKS102 | Staphylococcus hominis |
| 10 | CSF | TB case | CNS TB | (-) | Negative | M. tuberculosis |  | Propionibacterium acnes | Ralstonia solanacearum | Bradyrhizobium S23321 | Streptococcus oralis | Phanerochaete chrysosporium |
| 11 | CSF | TB case | CNS TB | (-) | Negative | M. tuberculosis |  | Burkholderia 383 | Bradyrhizobium japonicum | Ralstonia solanacearum | Propionibacterium acnes | Acidovorax KKS102 |
| 12 | CSF | TB case | CNS TB | (+) | Culture: M. tuberculosis | Aspergillus fumigatus |  | Staphylococcus epidermidis | Prevotella melaninogenica | Propionibacterium acnes | Acidovorax KKS102 |  |
| 13 | CSF | TB case | CNS TB | (-) | Negative |  |  | Brevundimonas subvibrioides | Propionibacterium acnes | Acidovorax KKS102 | Shamonda virus | Saccharomyces bayanus |
| 14 | CSF | TB case | CNS TB | (-) | Negative | M. tuberculosis |  | Asticcacaulis excentricus | Corynebacterium urealyticum | Phanerochaete chrysosporium |  |  |
| 15 | CSF | TB case | CNS TB | (-) | Culture: M. tuberculosis | M. tuberculosis | Staphylococcus aureus | Propionibacterium acnes | Achromobacter xylosoxidans | Methylobacterium radiotolerans | Micrococcus luteus | Pseudomonas putida |
| 16 | BALF | TB case | Disseminated TB | (-) | Negative |  |  | Propionibacterium acnes | Burkholderia 383 | Fusarium verticillioides | Malassezia globosa | Aspergillus terreus |
| 17 | BALF | TB case | Disseminated TB | (-) | Negative |  |  | Burkholderia 383 | Rothia mucilaginosa | Neisseria lactamica | Prevotella melaninogenica | Streptococcus parasanguinis |
| 18 | CSF | TB case | Disseminated TB | (-) | Negative |  |  | Burkholderia 383 | Propionibacterium acnes | Bradyrhizobium S23321 | Acidovorax KKS102 | Staphylococcus haemolyticus |
| 19 | Pus | TB case | Disseminated TB | (+) | Negative | M. tuberculosis |  |  |  |  |  |  |
| 20 | Sputum | TB case | Disseminated TB | (+) | Culture: M. tuberculosis |  |  | Prevotella melaninogenica | Veillonella parvula | Streptococcus infantis | Haemophilus parainfluenzae | Peptostreptococcus stomatis |
| 21 | BALF | TB case | Pulmonary TB | (-) | Negative |  |  | Prevotella melaninogenica | Rothia mucilaginosa | Haemophilus parainfluenzae | Veillonella parvula | Streptococcus oligofermentans |
| 22 | BALF | TB case | Pulmonary TB | (+) | Negative | M. tuberculosis |  | Prevotella intermedia | Burkholderia multivorans | Streptococcus anginosus | Propionibacterium acnes | Rothia mucilaginosa |
| 23 | BALF | TB case | Pulmonary TB | (+) | Culture: M. tuberculosis | M. tuberculosis |  | Prevotella melaninogenica | Streptococcus parasanguinis | Veillonella parvula | Rothia mucilaginosa | Atopobium parvulum |
| 24 | BALF | TB case | Pulmonary TB | (-) | Culture: M. tuberculosis |  |  | Prevotella melaninogenica | Streptococcus salivarius | Streptococcus parasanguinis | Alloprevotella tannerae | Neisseria mucosa |
| 25 | BALF | TB case | Pulmonary TB | (+) | Culture: M. tuberculosis | M. tuberculosis |  | Prevotella melaninogenica | Rothia mucilaginosa | Streptococcus parasanguinis | Porphyromonas gingivalis | Veillonella parvula |
| 26 | BALF | TB case | Pulmonary TB | (+) | Negative | M. tuberculosis |  | Porphyromonas gingivalis | Bradyrhizobium S23321 | Ralstonia solanacearum | Propionibacterium acnes | Tannerella forsythia |
| 27 | BALF | TB case | Pulmonary TB | (+) | Culture: M. tuberculosis | M. tuberculosis | Aspergillus oryzae | Malassezia globosa | Neisseria lactamica | Rothia mucilaginosa | Veillonella parvula | Lactobacillus fermentum |
| 28 | BALF | TB case | Pulmonary TB | (+) | Culture: M. tuberculosis | M. tuberculosis |  | Streptococcus salivarius | Veillonella parvula | Prevotella melaninogenica | Atopobium parvulum | Bifidobacterium dentium |
| 29 | BALF | TB case | Pulmonary TB | (-) | Negative | M. tuberculosis | Acinetobacter baumannii | Propionibacterium acnes | Prevotella melaninogenica | Veillonella parvula | Streptococcus I-P16 | Burkholderia multivorans |
| 30 | sputum | TB case | Pulmonary TB | (+) | Negative | M. tuberculosis |  | Corynebacterium accolens | Mycoplasma hominis | Brevundimonas diminuta |  |  |
| 31 | Pus | TB case | Soft tissue TB | (+) | Culture: M. tuberculosis | M. tuberculosis |  | Burkholderia 383 | Bradyrhizobium japonicum | Propionibacterium acnes | Methylobacterium radiotolerans | Ralstonia solanacearum |
| 32 | Pus | TB case | Soft tissue TB | (+) | Culture: M. tuberculosis |  |  | Propionibacterium acnes |  |  |  |  |
| 33 | Pus | TB case | Spinal TB | (+) | Pathology examination positive for Mtb | M. tuberculosis |  | Methanocorpusculum labreanum | Desulfarculus baarsii |  |  |  |
| 34 | Pus | TB case | TB lymhadenitis | (+) | Negative | M. tuberculosis |  | Enterobius vermicularissta | Trichinella zimbabwensis | Human herpesvirus 5 | Torque teno midi virus 2 |  |
| 35 | Ascites | TB case | TB Peritonitis | (-) | Negative |  |  | Acinetobacter johnsonii | Brevibacterium epidermidis | Mesorhizobium loti | Propionibacterium acnes | Slackia exigua |
| 36 | Ascites | TB case | TB Peritonitis | (-) | Negative |  |  | Burkholderia multivorans | Propionibacterium acnes | Bradyrhizobium japonicum | Ralstonia solanacearum | Acidovorax KKS102 |
| 37 | Pleural fluid | TB case | TB Peritonitis | (-) | Negative |  |  | Burkholderia multivorans | Ralstonia insidiosa | Propionibacterium acnes | Brevundimonas vesicularis | Pseudomonas mendocina |
| 38 | Ascites | TB case | TB Pleurisy | (+) | Negative |  |  | Prevotella intermedia | Porphyromonas gingivalis | Propionibacterium acnes | Corynebacterium aurimucosum | Rothia mucilaginosa |
| 39 | Pleural fluid | TB case | TB Pleurisy | (+) | Negative |  |  | Bradyrhizobium japonicum | Agromonas oligotrophica | Ralstonia solanacearum | Propionibacterium acnes | Methylobacterium radiotolerans |
| 40 | Pleural fluid | TB case | TB Pleurisy | (-) | Negative | Human herpesvirus 6A |  | Propionibacterium acnes | Bradyrhizobium japonicum | Micrococcus luteus | Human herpesvirus 6A | Phanerochaete chrysosporium |
| 41 | Pleural fluid | TB case | TB Pleurisy | (-) | Negative |  |  | Porphyromonas gingivalis | Prevotella melaninogenica | Propionibacterium acnes | Methanocorpusculum labreanum | Saccharomyces bayanus |
| 42 | Pleural fluid | TB case | TB Pleurisy | (-) | Negative | M. tuberculosis |  | Burkholderia cenocepacia | Propionibacterium acnes | Sphingopyxis alaskensis | Caulobacter crescentus | Methylobacterium chloromethanicum |
| 43 | Pleural fluid | TB case | TB Pleurisy | (+) | Negative |  |  | Bradyrhizobium japonicum | Burkholderia 383 | Acidovorax KKS102 | Methylobacterium radiotolerans | Propionibacterium acnes |
| 44 | Urine | TB case | Urinary tract TB | (-) | Negative |  |  | Staphylococcus epidermidis | Prevotella timonensis | Anaerococcus obesiensis | Propionibacterium acnes | Burkholderia multivorans |
| 45 | Urine | TB case | Urinary tract TB | (-) | Negative | Morganella morganii | Klebsiella pneumoniae | Burkholderia multivorans | Propionibacterium acnes | Shewanella MR |  |  |
| 46 | BALF | Non-TB case | Bacteria infection (atypical pneumonia) | (-) | Negative |  |  | Prevotella melaninogenica | Streptococcus parasanguinis | Streptococcus oralis | Rothia mucilaginosa | Neisseria meningitidis |
| 47 | Pleural fluid | Non-TB case | Bacteria infection (Invasion syndrome caused by Klebsiella pnuemoniae infection) | (-) | Negative | Klebsiella pneumoniae |  | Burkholderia multivorans | Ralstonia solanacearum | Lactobacillus plantarum | Streptococcus salivarius | Polaromonas naphthalenivorans |
| 48 | CSF | Non-TB case | Bacteria infection (purulent meningitis) | (-) | Negative |  |  | Bradyrhizobium japonicum | Methylobacterium radiotolerans | Acidovorax KKS102 | Propionibacterium acnes | Ralstonia solanacearum |
| 49 | CSF | Non-TB case | Bacteria infection (purulent meningitis) | (-) | Negative |  |  | Acidovorax KKS102 | Bradyrhizobium japonicum | Agromonas oligotrophica | Phanerochaete chrysosporium | Ochrobactrum anthropi |
| 50 | CSF | Non-TB case | Bacteria infection (purulent meningitis) | (-) | Negative |  |  | Propionibacterium acnes | Staphylococcus epidermidis | Corynebacterium diphtheriae | Acidovorax KKS102 | Malassezia globosa |
| 51 | CSF | Non-TB case | Bacteria infection (Streptococcus Infectious endocarditis) | (-) | Negative |  |  | Bradyrhizobium japonicum | Propionibacterium acnes | Ralstonia solanacearum | Bacillus amyloliquefaciens | Methylobacterium radiotolerans |
| 52 | Ascites | Non-TB case | Bacterial infection (Abdominal infection) | (-) | Negative | Pseudomonas aeruginosa | Enterococcus faecalis | Burkholderia 383 | Propionibacterium acnes | Bradyrhizobium BTAi1 | Staphylococcus epidermidis | Methylobacterium radiotolerans |
| 53 | Pus | Non-TB case | Bacterial infection (Bacteroides fragilis abcess) | (-) | Negative | Bacteroides fragilis |  | Tannerella forsythia | Alistipes shahii | Trichinella papuae |  |  |
| 54 | BALF | Non-TB case | Bacterial infection (M. abscessus pneumonia) | (-) | Negative | M. abscessus |  | Streptococcus oralis | Propionibacterium acnes | Rothia dentocariosa | Veillonella parvula | Staphylococcus haemolyticus |
| 55 | Pleural fluid | Non-TB case | Bacterial infection (Mycoplasma pneumoniae) | (-) | Negative |  |  | Ralstonia solanacearum | Bradyrhizobium japonicum | Propionibacterium acnes |  | Saccharomyces bayanus |
| 56 | BALF | Non-TB case | Bacterial infection (Pneumonia) | (-) | Negative | Enterococcus faecium | Human herpesvirus 4 | Methylobacterium radiotolerans | Burkholderia contaminans | Ralstonia insidiosa | Abiotrophia defectiva | Granulicatella adiacens |
| 57 | BALF | Non-TB case | Bacterial infection (Pneumonia) | (-) | Negative |  |  | Propionibacterium acnes | Streptococcus I-P16 | Burkholderia 383 | Rothia mucilaginosa | Veillonella parvula |
| 58 | sputum | Non-TB case | Bacterial infection (Pneumonia) | (-) | Negative | Pseudomonas aeruginosa |  | Pluralibacter gergoviae | Rothia mucilaginosa | Tannerella forsythia | Clostridium butyricum | Streptococcus infantis |
| 59 | CSF | Non-TB case | Bacterial infection (purulent meningitis) | (-) | Negative |  |  | Burkholderia multivorans | Propionibacterium acnes | Staphylococcus epidermidis | Micrococcus luteus | Corynebacterium aurimucosum |
| 60 | CSF | Non-TB case | Bacterial infection (purulent meningitis) | (-) | Negative |  |  | Methylobacterium radiotolerans | Propionibacterium acnes | Acidovorax KKS102 | Bradyrhizobium S23321 | Ralstonia solanacearum |
| 61 | CSF | Non-TB case | Bacterial infection (Scrub typhus) | (-) | Negative |  |  | Propionibacterium acnes | Acidovorax KKS102 | Burkholderia 383 | Bradyrhizobium japonicum | Pseudomonas aeruginosa |
| 62 | Pus | Non-TB case | Bacterial infection (Staphylococcus aureu abscess) | (-) | Culture: Staphylococcus aureus | Staphylococcus aureus |  | Ochrobactrum anthropi | Mesorhizobium australicum | Aspergillus flavus |  |  |
| 63 | BALF | Non-TB case | Bacterial infection (Staphylococcus aureu pneumonia) | (-) | Culture: Staphylococcus aureus | Haemophilus parainfluenzae | Staphylococcus aureus | Prevotella melaninogenica | Rothia mucilaginosa | Streptococcus oralis | Neisseria meningitidis | Veillonella parvula |
| 64 | CSF | Non-TB case | Fungal infection (Cryptococcal meningitis) | (-) | Negative | Cryptococcus neoformans |  | Burkholderia 383 | Bradyrhizobium S23321 | Propionibacterium acnes | Methylobacterium populi | Ralstonia solanacearum |
| 65 | CSF | Non-TB case | Fungal infection (Exophiala dermatitidis meningitis) | (-) | Negative |  |  | Burkholderia cepacia | Deinococcus geothermalis | Propionibacterium acnes | Streptococcus I-P16 | Methylobacterium radiotolerans |
| 66 | CSF | Non-TB case | Fungal infection (Fungal meningitis) | (-) | Negative |  |  | Propionibacterium acnes | Corynebacterium callunae | Staphylococcus capitis | Ralstonia solanacearum | Bradyrhizobium japonicum |
| 67 | BALF | Non-TB case | Fungal infection (Fungla pneumonia) | (-) | Negative | Human adenovirus B1 |  | Streptococcus parasanguinis | Prevotella melaninogenica | Haemophilus parainfluenzae | Rothia mucilaginosa | Neisseria lactamica |
| 68 | Pleural fluid | Non-TB case | Fungal infection (Pneumonia Coccidioides immitis) | (-) | Negative | Coccidioides immitis |  | Propionibacterium acnes | Ralstonia solanacearum | Rothia mucilaginosa | Methylobacterium chloromethanicum | Acidovorax JS42 |
| 69 | BALF | Non-TB case | Fungal infection (Pulmonary aspergillosis) | (-) | Pathology examination positive for Aspergillus |  |  | Micrococcus luteus | Bradyrhizobium japonicum | Propionibacterium acnes | Agromonas oligotrophica | Phanerochaete chrysosporium |
| 70 | BALF | Non-TB case | Fungal infection(Fungal Pneumonia) | (-) | Negative | Human herpesvirus 1 |  | Streptococcus salivarius | Propionibacterium acnes | Prevotella melaninogenica | Veillonella parvula | Rothia mucilaginosa |
| 71 | BALF | Non-TB case | Fungal infection(Fungal Pneumonia) | (-) | Negative |  |  | Prevotella melaninogenica | Streptococcus I-P16 | Rothia mucilaginosa | Veillonella parvula | Campylobacter concisus |
| 72 | CSF | Non-TB case | Malignancies (CNS lymphoma) | (-) | Negative |  |  | Burkholderia 383 | Propionibacterium acnes | Ralstonia solanacearum | Micrococcus luteus | Staphylococcus epidermidis |
| 73 | CSF | Non-TB case | Malignancies (CNS tumor) | (-) | Negative |  |  | Burkholderia multivorans | Acidovorax KKS102 | Propionibacterium acnes | Bradyrhizobium japonicum | Streptococcus oralis |
| 74 | CSF | Non-TB case | Malignancies (CNS tumor) | (-) | Negative |  |  | Burkholderia 383 | Propionibacterium acnes | Agromonas oligotrophica | Ralstonia solanacearum | Acidovorax KKS102 |
| 75 | CSF | Non-TB case | Malignancies (CNS tumor) | (-) | Negative |  |  | Burkholderia 383 | Propionibacterium acnes | Staphylococcus epidermidis | Cupriavidus metallidurans | Acinetobacter lwoffii |
| 76 | CSF | Non-TB case | Malignancies (CNS tumor) | (-) | Negative |  |  | Burkholderia ubonensis | Propionibacterium acnes | Staphylococcus epidermidis | Rhodococcus erythropolis | Oscillatoria PCC |
| 77 | BALF | Non-TB case | Malignancies (MALT lymphoma) | (-) | Negative |  |  | Streptococcus intermedius | Rothia mucilaginosa | Neisseria meningitidis | Thermus scotoductus | Propionibacterium acnes |
| 78 | Ascites | Non-TB case | Malignancies (Non Hodgin's lymphoma) | (-) | Negative |  |  | Prevotella pallens | Neisseria flavescens | Propionibacterium acnes | Staphylococcus haemolyticus | Burkholderia contaminans |
| 79 | Pleural fluid | Non-TB case | Malignancies (Non Hodgin's lymphoma) | (-) | Negative |  |  | Stenotrophomonas maltophilia | Escherichia fergusonii | Human herpesvirus 4 | Lachancea waltii | Meyerozyma guilliermondii |
| 80 | Lung tissue | Non-TB case | Miscellaneous causes (Cryptogenic Organizing Pneumonia) | (-) | Negative |  |  | Propionibacterium acnes | Burkholderia 383 | Ralstonia solanacearum | Bradyrhizobium japonicum | Staphylococcus hominis |
| 81 | Ascites | Non-TB case | Miscellaneous causes (Decompensentated liver cirrhosis) | (-) | Negative |  |  | Burkholderia multivorans | Bradyrhizobium BTAi1 | Propionibacterium acnes | Prevotella intermedia | Methylobacterium radiotolerans |
| 82 | Pleural fluid | Non-TB case | Miscellaneous causes (Decompensentated liver cirrhosis) | (-) | Negative |  |  | Propionibacterium acnes | Staphylococcus haemolyticus | Brevundimonas subvibrioides | Asticcacaulis excentricus | Saccharomyces bayanus |
| 83 | CSF | Non-TB case | Miscellaneous causes (Hypertrophic cranial pachymeningitis) | (-) | Negative |  |  | Burkholderia multivorans | Methylobacterium radiotolerans | Propionibacterium acnes | Staphylococcus epidermidis | Micrococcus luteus |
| 84 | CSF | Non-TB case | Miscellaneous causes (Hypertrophic cranial pachymeningitis) | (-) | Negative |  |  | Bradyrhizobium S23321 | Propionibacterium acnes | Burkholderia 383 | Methylobacterium radiotolerans | Malassezia globosa |
| 85 | BALF | Non-TB case | Miscellaneous causes (Lung nodule) | (-) | Negative |  |  | Prevotella melaninogenica | Veillonella parvula | Streptococcus parasanguinis | Campylobacter concisus | Corynebacterium argentoratense |
| 86 | CSF | Non-TB case | Miscellaneous causes (Possibly CNS tumor) | (-) | Negative |  |  | Propionibacterium acnes | Bradyrhizobium S23321 | Acidovorax KKS102 | Staphylococcus epidermidis | Corynebacterium callunae |
| 87 | CSF | Non-TB case | Miscellaneous causes (Possibly CNS tumor) | (-) | Negative |  |  | Propionibacterium acnes | Bradyrhizobium S23321 | Acidovorax KKS102 | Agromonas oligotrophica | Staphylococcus epidermidis |
| 88 | Ascites | Non-TB case | Miscellaneous causes (Primary peritonitis) | (-) | Negative |  |  | Corynebacterium matruchotii | Mesorhizobium australicum | Rhodoferax ferrireducens | Hyphomonas polymorpha | Actinomyces viscosus |
| 89 | Joint Fluids | Non-TB case | Miscellaneous causes (Reactive arthritis) | (-) | Negative |  |  | Burkholderia contaminans | Ralstonia insidiosa | Bradyrhizobium BTAi1 | Acinetobacter johnsonii | Actinomyces georgiae |
| 90 | CSF | Non-TB case | Noninfectious inflammatory disease (acute disseminated encephalomyelitis) | (-) | Negative |  |  | Burkholderia 383 | Propionibacterium acnes | Bradyrhizobium japonicum | Ralstonia solanacearum | Micrococcus luteus |
| 91 | CSF | Non-TB case | Noninfectious inflammatory disease (Autoimmune encephalitis) | (-) | Negative |  |  | Rothia dentocariosa | Prevotella intermedia | Staphylococcus lugdunensis | Streptococcus mitis | Porphyromonas gingivalis |
| 92 | CSF | Non-TB case | Noninfectious inflammatory disease (Autoimmune encephalitis) | (-) | Negative |  |  | Propionibacterium acnes | Bradyrhizobium japonicum | Methylobacterium radiotolerans | Aspergillus flavus | Phanerochaete chrysosporium |
| 93 | CSF | Non-TB case | Noninfectious inflammatory disease (Autoimmune encephalitis) | (-) | Negative | M. tuberculosis |  | Propionibacterium acnes | Staphylococcus capitis | Corynebacterium callunae | Brevundimonas subvibrioides | Malassezia globosa |
| 94 | CSF | Non-TB case | Noninfectious inflammatory disease (Guillain-Barre syndrome) | (-) | Negative |  |  | Propionibacterium acnes | Streptococcus sanguinis | Acidovorax KKS102 | Staphylococcus warneri | Bradyrhizobium japonicum |
| 95 | CSF | Non-TB case | Noninfectious inflammatory disease (IgG4-related disease) | (-) | Negative |  |  | Propionibacterium acnes | Burkholderia multivorans | Bradyrhizobium japonicum | Staphylococcus epidermidis | Streptococcus oralis |
| 96 | Ascites | Non-TB case | Noninfectious inflammatory disease (Vasculitis) | (-) | Culture：Enterococcus gallinarum |  |  | Saccharomyces bayanus |  |  |  |  |
| 97 | CSF | Non-TB case | Viral infection (Viral encephalitis) | (-) | Negative | Human herpesvirus 3 |  | Propionibacterium acnes | Bradyrhizobium S23321 | Ralstonia solanacearum | Bradyrhizobium japonicum | Corynebacterium callunae |
| 98 | CSF | Non-TB case | Viral infection (Viral encephalitis) | (-) | Negative | Human adenovirus B1 |  | Propionibacterium acnes | Micrococcus luteus | Bradyrhizobium S23321 | Staphylococcus epidermidis | Brevundimonas subvibrioides |
| 99 | CSF | Non-TB case | Viral infection (Viral encephalitis) | (-) | Negative |  |  | Propionibacterium acnes | Brevundimonas subvibrioides | Acidovorax KKS102 | Saccharomyces bayanus | Staphylococcus epidermidis |
| 100 | CSF | Non-TB case | Viral infection (Viral encephalitis) | (-) | Negative | Human herpesvirus 3 |  | Burkholderia multivorans | Propionibacterium acnes | Bradyrhizobium japonicum | Pseudomonas TKP | Ralstonia solanacearum |
| 101 | CSF | Non-TB case | Viral infection (Viral encephalitis) | (-) | Negative | Human herpesvirus 3 |  | Burkholderia 383 | Propionibacterium acnes | Acidovorax KKS102 | Cupriavidus metallidurans | Ralstonia solanacearum |
| 102 | CSF | Non-TB case | Viral infection (Viral encephalitis) | (-) | Negative |  |  | Burkholderia multivorans | Propionibacterium acnes | Bradyrhizobium japonicum | Staphylococcus epidermidis | Acidovorax KKS102 |
| 103 | CSF | Non-TB case | Viral infection (Viral encephalitis) | (-) | Negative |  |  | Propionibacterium acnes | Bradyrhizobium S23321 | Staphylococcus epidermidis | Brevundimonas subvibrioides | Phanerochaete chrysosporium |
| 104 | CSF | Non-TB case | Viral infection (Viral encephalitis) | (-) | Negative |  |  | Burkholderia contaminans | Ralstonia insidiosa | Propionibacterium acnes | Bradyrhizobium BTAi1 | Acidovorax KKS102 |
| 105 | CSF | Non-TB case | Viral infection (Viral encephalitis) | (-) | Negative |  |  | Propionibacterium acnes | Acidovorax KKS102 | Bradyrhizobium japonicum | Ralstonia solanacearum | Staphylococcus epidermidis |

**Table S2.** Correlation of mNGS assay versus culture and Xpert.

|  |  |  | Culture | | Xpert | |
| --- | --- | --- | --- | --- | --- | --- |
|  |  |  | + | - | + | - |
| All samples | mNGS | + | 10 | 11 | 12 | 9 |
|  |  | - | 3 | 81 | 7 | 77 |
| Pulmonary samples | mNGS | + | 4 | 4 | 7 | 1 |
|  |  | - | 1 | 18 | 1 | 18 |
| CSF samples | mNGS | + | 2 | 6 | 1 | 7 |
|  |  | - | 1 | 40 | 2 | 39 |
| Other extrapulmonary samples | mNGS | + | 4 | 1 | 4 | 1 |
|  |  | - | 1 | 23 | 4 | 20 |
