# Supplementary figures and images for: Clinical Evaluation of Diagnosis Efficacy of Active Mycobacterium tuberculosis Complex Infection via Metagenomic Next-Generation Sequencing of Direct Clinical Samples
Source: Front Cell Infect Microbiol. 2019 Oct 18;9:351. doi: 10.3389/fcimb.2019.00351 (PMC6813183; doi:10.3389/fcimb.2019.00351)

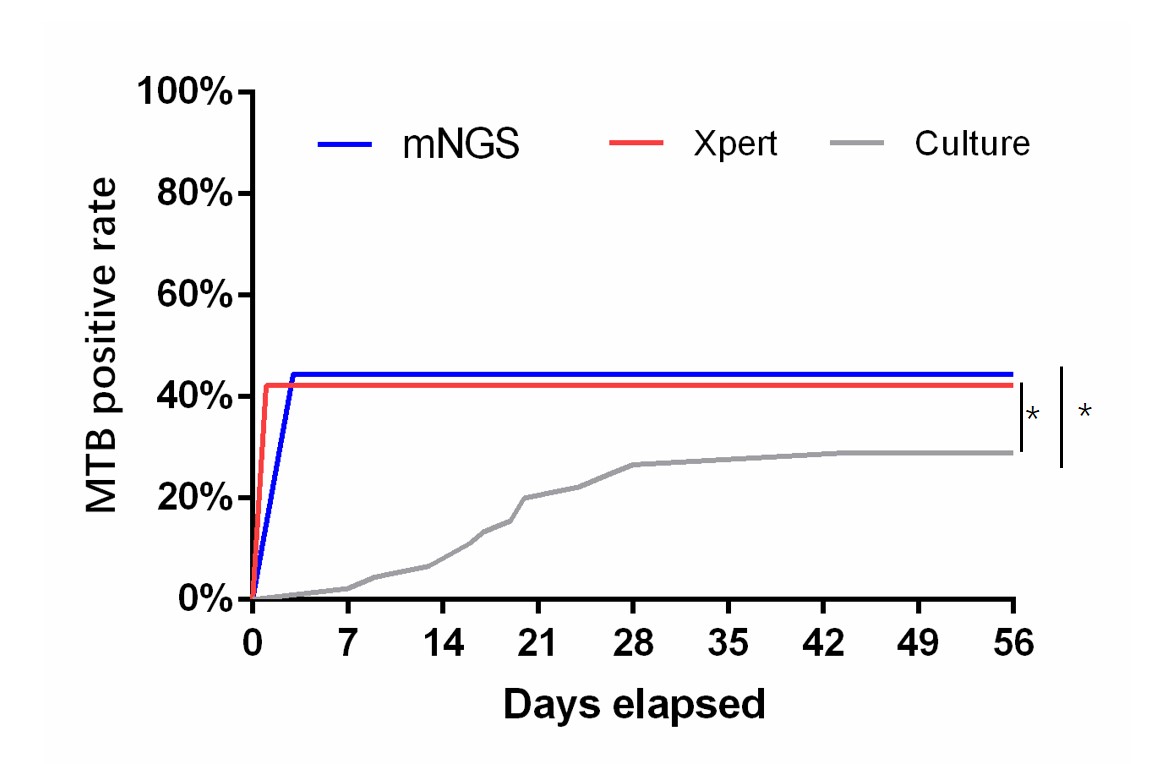

Supplement: Supplementary file 2 [file Image_1.JPEG]
